# Correct Me if I'm Wrong: Groups Outperform Individuals in the Climate Stabilization Task

**DOI:** 10.3389/fpsyg.2018.02274

**Published:** 2018-11-30

**Authors:** Belinda Xie, Mark J. Hurlstone, Iain Walker

**Affiliations:** ^1^School of Psychological Science, University of Western Australia, Crawley, WA, Australia; ^2^School of Psychology, University of New South Wales, Sydney, NSW, Australia; ^3^School of Psychology, University of Canberra, Bruce, ACT, Australia

**Keywords:** climate stabilization task, mental models, group decision-making, carbon dioxide accumulation, stock-flow tasks, emissions reduction

## Abstract

Avoiding dangerous climate change requires ambitious emissions reduction. Scientists agree on this, but policy-makers and citizens do not. This discrepancy can be partly attributed to faulty mental models, which cause individuals to misunderstand the carbon dioxide (CO_2_) system. For example, in the Climate Stabilization Task (hereafter, “CST”) (Sterman and Booth-Sweeney, 2007), individuals systematically underestimate the emissions reduction required to stabilize atmospheric CO_2_ levels, which may lead them to endorse ineffective “wait-and-see” climate policies. Thus far, interventions to correct faulty mental models in the CST have failed to produce robust improvements in decision-making. Here, in the first study to test a group-based intervention, we found that success rates on the CST markedly increased after participants deliberated with peers in a group discussion. The group discussion served to invalidate the faulty reasoning strategies used by some individual group members, thus increasing the proportion of group members who possessed the correct mental model of the CO_2_ system. Our findings suggest that policy-making and public education would benefit from group-based practices.

## Introduction

To avoid dangerous climate change, average global temperature must not exceed a critical threshold, defined in the Paris Agreement as 1.5–2°C above pre-industrial levels (UNFCCC, 2015). However, countries' current climate pledges are guaranteed to overshoot this threshold (Mauritsen and Pincus, 2017), indicating that current national emissions policies are grossly inadequate. In a democracy, implementing effective mitigation policy is a two-step challenge: policy-makers must craft appropriate policies and those policies must then receive political and electoral support (Dreyer et al., 2015). Both steps require policy-makers, politicians, and citizens to understand the CO_2_ system. Unfortunately, most individuals lack this knowledge and consequently underestimate the measures required to mitigate climate change (e.g., Sterman and Booth-Sweeney, 2002; Martin, 2008; Guy et al., 2013).

To reason about emissions policy (in the context of mitigating climate change), an individual must understand how CO_2_ emissions contribute to climate change. For example, someone who accepts the scientific consensus would: (1) recognize that global temperature is increasing, (2) attribute that increase to human CO_2_ emissions, and (3) predict that emitting more CO_2_ will further increase temperature. This knowledge structure is called a “mental model” (Sterman, 1994; Doyle and Ford, 1998). A mental model represents the causal relationships within a system, and is used to describe, explain, and predict system behavior (Sterman, 1994; Doyle and Ford, 1998). Although crucial for decision-making, mental models are constrained by cognitive limits (Doyle and Ford, 1998; Sterman and Booth-Sweeney, 2002) and can never represent the full complexity of the real world. The human decision-maker is thus likely to make imperfect decisions about complex problems.

**Figure 1:**
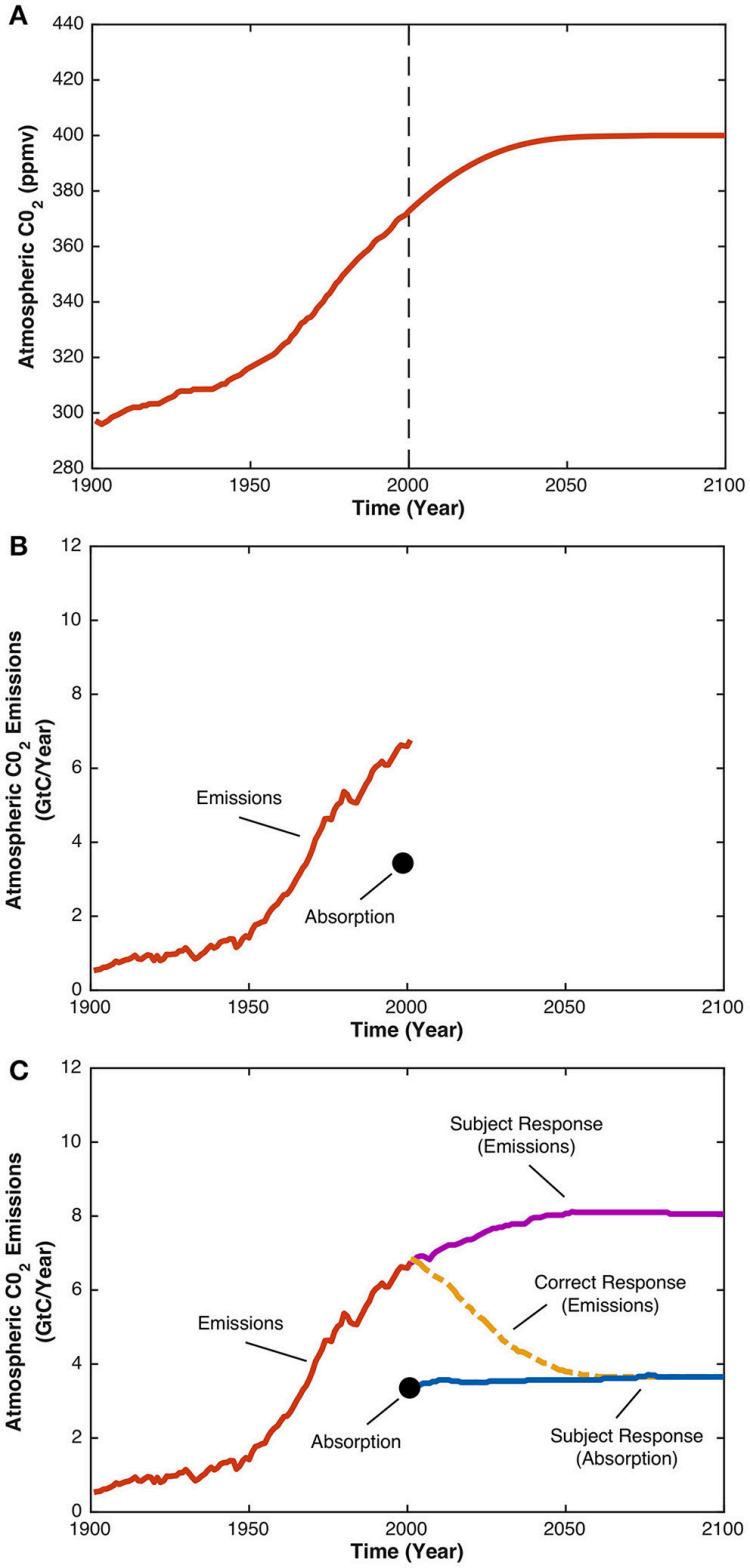
Graphical illustration of the CST. Participants are presented with the graph in **(A)** showing the increase of atmospheric CO_2_ concentration since the year 1900 up until the year 2000. Following 2000, the graph depicts a hypothetical scenario in which atmospheric CO_2_ concentration increases to 400 ppm before stabilizing by the year 2100. Next, participants are presented with the graph in **(B)** and asked to sketch the trajectories of CO_2_ emissions and CO_2_ absorption from years 2000 to 2100 that they believe would be consistent with the hypothetical scenario. The graph in **(C)** shows a typical participant's response to the CST, where the blue line represents the participant's estimate of CO_2_ absorption, and the purple line represents the participant's estimate of CO_2_ emissions. As the rate of CO_2_ emissions exceeds the rate of CO_2_ absorption, atmospheric CO_2_ concentration will increase, not stabilize. This is an example of the so-called “pattern-matching” heuristic, whereby the pattern of CO_2_ emissions is assumed to “match” the pattern of atmospheric CO_2_ concentration. The correct CO_2_ emissions trajectory, given the participant's estimate of CO_2_ absorption, is depicted by the dashed yellow line. The rate of CO_2_ emissions decreases to equal the rate of CO_2_ absorption, an equilibrium that would stabilize atmospheric CO_2_ concentration. This response is consistent with the principle of accumulation, which states that the level of a stock at any given time is the difference between its inflow and its outflow.

The Climate Stabilization Task (hereafter, “CST”) represents the complex problem of choosing the appropriate level of climate change mitigation. The CST is a decision-making task in which participants are told that atmospheric CO_2_ concentration is increased by CO_2_ emissions (largely from human activities), decreased by CO_2_ absorption (largely by oceans and plants), and stabilized when the rate of CO_2_ emissions equals the rate of CO_2_ absorption. Participants are also told that atmospheric CO_2_ concentration has increased since the Industrial Revolution, because the rate of CO_2_ emissions has increased to double the rate of CO_2_ absorption. Participants are then presented with a hypothetical scenario (Figure 1A) in which atmospheric CO_2_ concentration gradually rises to 400 ppm, then stabilizes by the year 2100. Next, participants must sketch trajectories of CO_2_ emissions and CO_2_ absorption that would correspond with this hypothetical scenario (Figure 1B).

The “principle of accumulation” states that, at any given time, the level of some accumulating stock (in this case, atmospheric CO_2_ concentration) is the difference between its inflow (rate of CO_2_ emissions) and outflow (rate of CO_2_ absorption) (Martin, 2008). Thus, to stabilize atmospheric CO_2_ concentration, the rate of CO_2_ emissions must decrease to equal the rate of CO_2_ absorption. However, participants often erroneously assert that stabilizing CO_2_ emissions is sufficient to stabilize atmospheric CO_2_ concentration (Figure 1C). Known as “pattern-matching,” this occurs when participants ignore CO_2_ absorption, believing that the pattern of atmospheric CO_2_ concentration should “match” the pattern of CO_2_ emissions. Repeated studies find that only 6–44% of participants answer the CST correctly, with many falling prey to the above mentioned pattern-matching heuristic (Sterman and Booth-Sweeney, 2002, 2007; Moxnes and Saysel, 2009; Boschetti et al., 2012; Guy et al., 2013; Newell et al., 2015). Low success rates on the CST are observed not only for members of the general public (Boschetti et al., 2012), but also for individuals who are a good proxy for policy-makers—namely stakeholders of a project researching climate change impacts (Boschetti et al., 2012) and Masters students studying system dynamics at the Massachusetts Institute of Technology (Sterman and Booth-Sweeney, 2002).

Most interventions to correct decision-makers' mental models of the CO_2_ system—as indexed by responses on the CST—have been unsuccessful (e.g., Pala and Vennix, 2005; Reichert et al., 2015). Using analogies (e.g., a bathtub in which the water level represents atmospheric CO_2_ concentration) (Moxnes and Saysel, 2009; Guy et al., 2013; Newell et al., 2015) and promoting “global thinking” over “local thinking” (Fischer and Gonzalez, 2015; Weinhardt et al., 2015) have produced minor improvements in CST performance. A formal university course in system dynamics was more successful (Pala and Vennix, 2005; Sterman, 2010), but this intervention is too resource-intensive to be applied on a large scale. Although these results seem discouraging, all interventions so far share the limitation of characterizing decision-makers as individuals. However, real-world decision-making is a social, group-based process informed by the beliefs of others (Tranter, 2011). Previous research shows that groups are better able than individuals to attenuate cognitive biases and decision heuristics (Kugler et al., 2012; Schulze and Newell, 2016), as well as identify, evaluate, and resolve competing hypotheses (Trouche et al., 2015; Larrick, 2016). These benefits notwithstanding, it is important to note that groups do not outperform individuals in all tasks. However, groups do perform consistently better than individuals on intellective, “truth-wins” problems in which the sole correct answer can be determined through logic, and then explained to convince others (i.e., the truth “wins”) (Davis, 1973; Laughlin et al., 2006). The CST is one such problem, as understanding the principle of accumulation leads to only one demonstrably correct solution (i.e., the rate of emissions equaling the rate of absorption).

**Figure 2:**
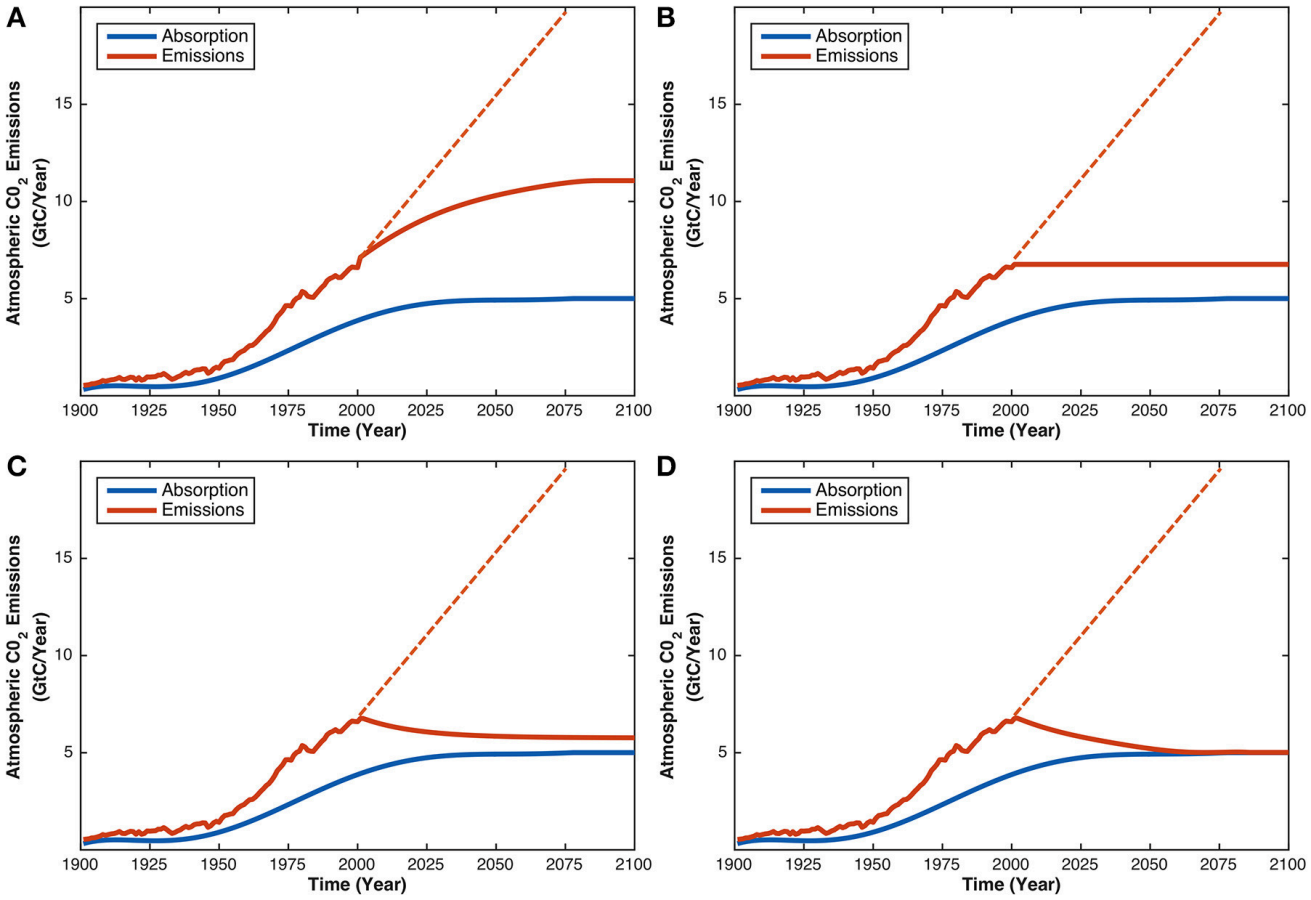
The four response alternatives in the multiple-choice version of the CST. All graphs show the same CO_2_ absorption trajectory with a different CO_2_ emissions trajectory. Graph **(A)** depicts the typical pattern-matching response in which CO_2_ emissions rise and then stabilize. Graph **(B)** is a less obvious form of pattern-matching in which CO_2_ emissions immediately stabilize. Graph **(C)** approximates the correct answer as CO_2_ emissions decrease, but not to the level required to achieve stabilization. Graph **(D)** is the correct response, because CO_2_ emissions decrease to equal CO_2_ absorption, thus stabilizing atmospheric CO_2_.

The aim of the current study was to test whether an intervention involving group decision-making can improve performance on the CST. To address this question, we administered a computerized version of the CST to staff and students from the University of Western Australia (*N* = 141). Participants were given background information about the CO_2_ system, and then presented with the hypothetical scenario in which atmospheric CO_2_ concentration stabilizes by the year 2100 (Figure 1A). The decision-making component was administered at two time points, Time 1 (*T*_1_) and Time 2 (*T*_2_). At *T*_1_, participants were presented with four graphs (Figure 2) and asked to select the graph that would produce the hypothetical scenario described. After selecting a graph, participants typed a brief explanation for their decision. At *T*_2_, participants were randomly allocated to one of three experimental conditions, before entering an anonymized online chatroom for 10 minutes. In the individuals condition (*N* = 21), participants reflected on their initial answer and explanation by themselves before making a decision about the four graphs again. In the dyads (*N* = 40) and groups (*N* = 80) conditions, either two or four participants, respectively, inspected each other's initial answers and explanations, then engaged in a discussion to reach a consensus decision on which of the four graphs is correct.

There were three key predictions. Firstly, it was predicted that the individual reflection would have no effect on decision-making, such that the success rates of individuals would not increase from *T*_1_ to *T*_2_. Secondly, it was predicted that the dyad discussion would benefit decision-making, such that the success rates of dyads would increase from *T*_1_ to *T*_2_. Thirdly, it was predicted that the group discussion would benefit decision-making to a greater extent than the dyad discussion, such that the success rates of groups would increase from *T*_1_ to *T*_2_, and this increase would be greater than that observed for dyads.

A secondary aim of the current study was to examine whether individual performance on the CST can be explained by a person's (1) demographic characteristics, (2) climate change knowledge and attitudes, and/or (3) personality and cognitive style. These constructs influence performance on comparable tasks that tap similar reasoning skills, but their effects on CST performance are unclear. Participants therefore completed a pre-test questionnaire assessing several individual differences measures. This was an exploratory feature of the current study and accordingly we made no specific predictions about the relationships between the following variables and CST performance.

Most studies find no relationship between demographic variables and performance on tasks similar to the CST (e.g., Moxnes and Saysel, 2004; Sterman, 2008). However, other studies find that younger participants (Browne and Compston, 2015), males (Ossimitz, 2002; Browne and Compston, 2015; Reichert et al., 2015), or students studying STEM degrees (Booth-Sweeney and Sterman, 2000; Browne and Compston, 2015) perform better than older participants, females, or students studying non-STEM degrees. Age, sex, and field of education were therefore included in the questionnaire.

The questionnaire also included a measure of “climate change knowledge,” as task-specific knowledge is associated with better performance on some stock-flow tasks (Strohhecker and Größler, 2013), but appears unrelated to performance on the CST (Moxnes and Saysel, 2004). A measure of “climate change attitudes” was also included to rule out the possibility that participants choose a graph based on their own ideology, rather than stock-flow reasoning. For the same reason, a measure of “environmental worldview,” or one's beliefs about humanity's relationship with nature (Price et al., 2014), was also included.

Two personality variables are related to the ability to overcome bias by prior belief (Homan et al., 2008; West et al., 2008), and may therefore benefit performance on the CST. “Active open-mindedness” describes an individual's tendency to spend sufficient time on a problem before giving up, and to consider new evidence and the beliefs of others (Haran et al., 2013). “Need for cognition” is the psychological need to structure the world in meaningful and integrated ways, and is associated with expending greater mental effort and enjoying analytical activity (Cacioppo and Petty, 1982).

Lastly, three aspects of cognitive style may be relevant to task performance. “Cognitive reflection” is the ability to resist reporting the first answer that comes to mind (Frederick, 2005), and may therefore protect against pattern-matching. “Global processing” is a way of perceiving the world that favors the organized whole, whereas “local processing” favors component parts and details (Weinhardt et al., 2015). Previous studies have produced conflicting results on the relationship between processing style and stock-flow reasoning (Fischer and Gonzalez, 2015; Weinhardt et al., 2015). “Systems thinking” refers to the tendency to understand phenomena as emerging from complex, dynamic, and nested systems (Thibodeau et al., 2016). It is positively related to the ability to comprehend causal complexity and dynamic relationships (Thibodeau et al., 2016), as well as pro-environmental attitudes (Davis and Stroink, 2016; Lezak and Thibodeau, 2016).

A third and final aim relates to Sterman's (2008) contention that the widespread, global preference for “wait-and-see” or “go-slow” approaches to emissions reduction can be linked to misunderstanding the complex CO_2_ system. We therefore included a policy preference question in the pre-test questionnaire, which was subsequently repeated at post-test, after completion of the CST. Participants answered the question, “Which of these comes closest to your view on how we should address climate change?” with one of three options: “wait-and-see” (wait until we are sure that climate change is really a problem before taking significant economic action), “go-slow” (we should take low-cost action as climate change effects will be gradual), or “act-now” (climate change is a serious and pressing problem that requires significant action now). If poor understanding of the climate system is indeed responsible for complacent attitudes toward emissions reduction, then we expect participants who answer the CST incorrectly to be more likely to prefer “wait-and-see” or “go-slow” policies at post-test. Conversely, those who answer the CST correctly should be more likely to select the “act-now” option.

## Method

Ethical approval to conduct the experiment was granted by the Human Ethics Office at the University of Western Australia (UWA) (RA/4/1/6298).

### Participants

One hundred and forty one members of the campus community at the UWA were recruited to take part in the experiment using the Online Recruitment System for Economic Experiments (ORSEE; Greiner, 2015), an open-source web-based recruitment platform for running decision-making experiments. The ORSEE database at UWA contains a pool of over 1,500 staff and students from a range of academic disciplines. Participants were recruited by issuing electronic invitations to randomly selected individuals in the ORSEE database to attend one of several advertised experimental sessions. Participants' ages ranged from 17 to 74 (*Mdn* = 21.00, *M* = 23.80, *SD* = 7.34) and just over two thirds of participants were female (69.5%). About half studied a degree-specific major under the Faculty of Science (54.5%), but the Business School (15.7%), Engineering, Computing, and Mathematics (14.2%), and Arts (13.4%) faculties were also well represented. Participants were paid $10AUD for attending the experiment.

### Design

The experiment manipulated two independent variables: group size (individuals [*n* = 1] vs. dyads [*n* = 2] vs. groups [*n* = 4]) and time (*T*_1_ vs. *T*_2_). Group size was a between-participants variable, whereas time was a within-participants variable. Participants were allocated to the different group size conditions in a quasi-random fashion (see below). There was a minimum of 20 cases per group size: 21 × 1 = 21 participants in the individuals condition; 20 × 2 = 40 participants in the dyads condition; and 20 × 4 = 80 participants in the groups condition.

### Apparatus, materials, and procedure

The experiment was conducted between May and August 2016 in the Behavioral Economics Laboratory at the UWA (http://bel-uwa.github.io), a computerized laboratory designed for carrying out collective decision-making experiments. There were 27 experimental sessions in total, with a minimum of two and a maximum of eight participants per session. Group sizes were randomly pre-determined before each session but were subject to change in the event that some participants failed to attend. For example, if eight participants were invited to a session, the goal was often to run two groups of four participants. If however, only six participants attended, then four participants were allocated to the group condition, and two participants were allocated at random either to the dyads condition or the individuals condition.

As participants arrived to each experimental session, they were randomly seated at a workstation containing two computer terminals. This random seating allocation in turn determined the group size condition to which the participants were allocated. The workstations were separated from each other by privacy blinds to prevent participants from observing one another's responses, and participants knew that face-to-face communication was prohibited. Participants read an information sheet and provided written informed consent, after which the experimenter provided an overview of the structure of the session. Using the left computer terminal on their workstation, the participants then completed the individual differences questionnaire (see Supplementary Materials, Section Individual Differences Questionnaire), which was executed on an internet browser using Qualtrics survey software. The questionnaire took approximately 20 minutes to complete.

Once all participants had completed the questionnaire, they received verbal instructions from the experimenter to minimize their internet browser, which revealed the electronic instructions for the CST (see Supplementary Materials, Section Instructions for CST). The first page foreshadowed what the task would involve. The second page defined CO_2_, CO_2_ emissions, atmospheric CO_2_ concentration, and CO_2_ absorption. The third page described how CO_2_ emissions and CO_2_ absorption, respectively, increase and decrease atmospheric CO_2_ concentration, and why atmospheric CO_2_ concentration has increased since the Industrial Revolution. The final page presented the decision-making situation. It described a hypothetical scenario in which atmospheric concentration rises from its current level of 400 ppm to stabilize at 420 ppm by the year 2100. Participants were then confronted with four graphs depicting the same trajectory of CO_2_ absorption, but different trajectories of CO_2_ emissions (Figure 2), and were required to choose the graph that would give rise to the hypothetical scenario. Graph D (Figure 2D) is the correct response, as it is the only graph that depicts the rate of CO_2_ emissions decreasing to equal the rate of CO_2_ absorption. We used a multiple-choice format because it is less cognitively-taxing than the version of the CST in which participants sketch trajectories. In Sterman and Booth-Sweeney (2007), a multiple-choice condition with seven textual response alternatives produced equivalent results to conditions requiring participants to sketch graphs.

The decision-making component of the CST was executed as a z-Tree (Fischbacher, 2007) program, which was administered on the right computer terminal of each participant's workstation. The CST required a decision at two different points in time: *T*_1_ and *T*_2_. At *T*_1_, all participants completed the task individually, irrespective of the group size condition to which they had been allocated. The experimental procedure at this time point was therefore identical across all three group size conditions. Participants first read the electronic instructions on the left computer terminal, before indicating on the right computer terminal which of the four graphs they believed would stabilize atmospheric CO_2_ concentration (Figure 2). There was no time limit for this component of the task.

Once participants had registered their *T*_1_ graph choice, a text field appeared on screen with a prompt to use the keyboard to type out a brief explanation for why they thought that the graph they had chosen was correct. Participants were allocated 5 minutes to complete this component of the task and a counter in the top right-hand corner of the terminal display indicated the time remaining for participants to supply their written explanations.

**Figure 3:**
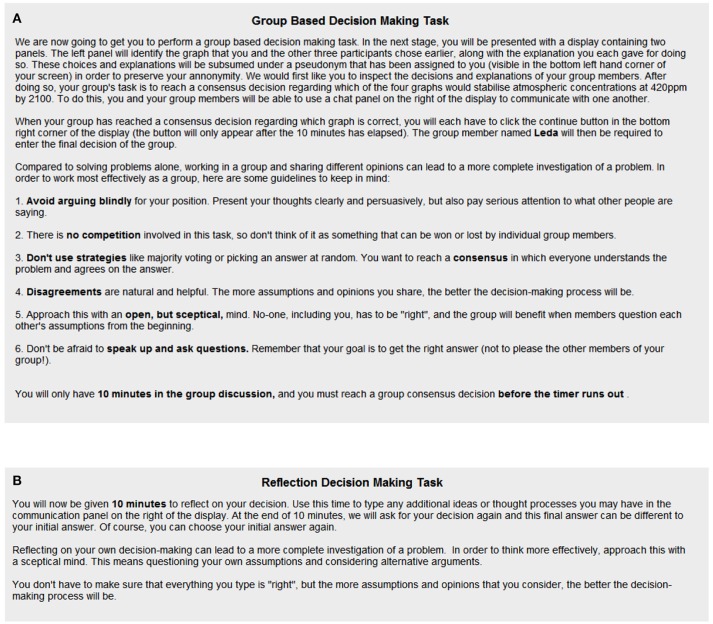
The instructions given to participants in the groups and dyads conditions **(A)** and individuals condition **(B)** at *T*_2_.

**Figure 4:**
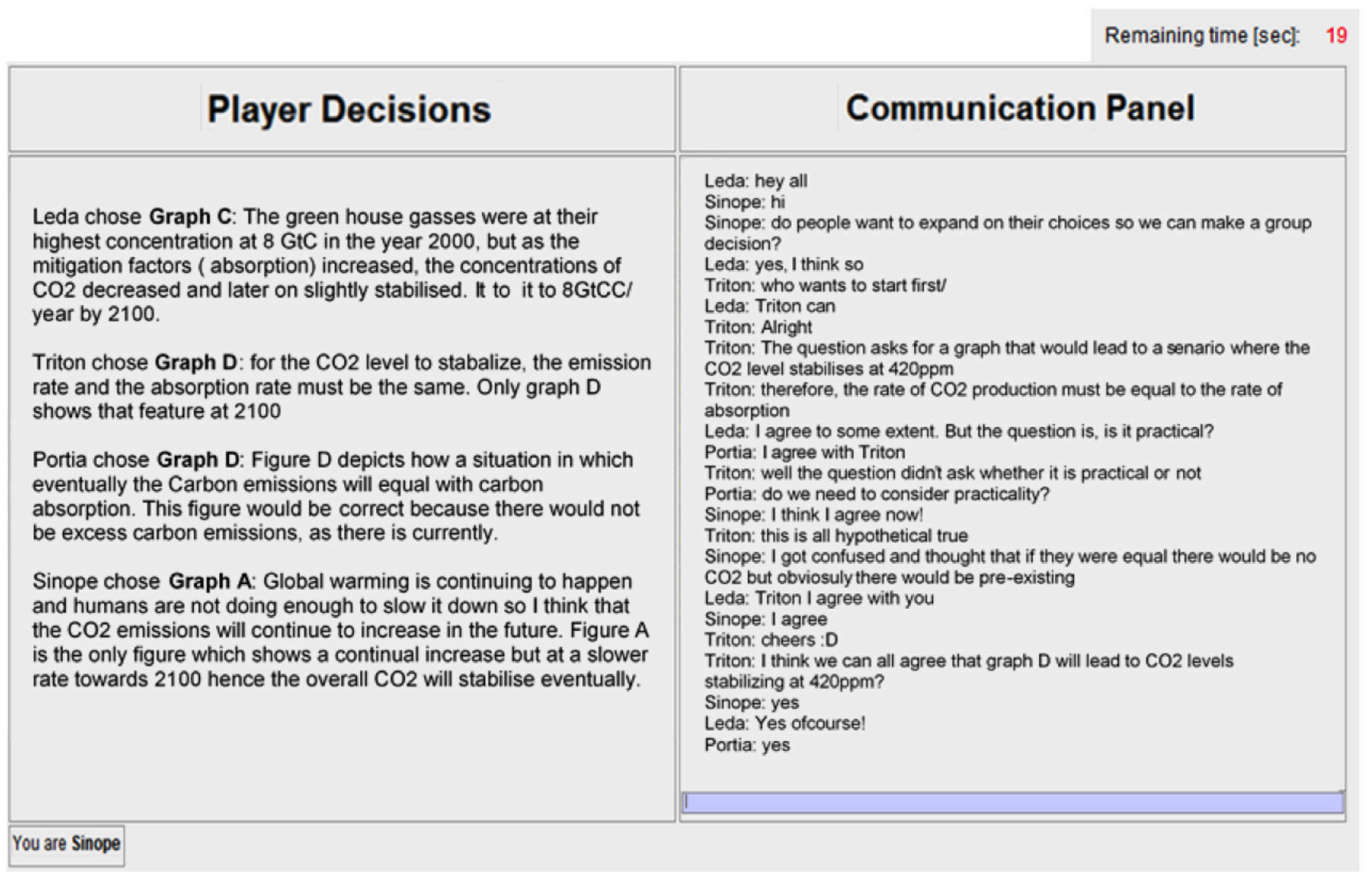
Graphical illustration of the chatroom communication interface.

At *T*_2_, participants were informed of their group size condition allocation. Participants assigned to the dyads or groups conditions were required to discuss the decision problem with their one partner or three group members, respectively, for a fixed period of 10 minutes in order to reach a consensus decision regarding the correct solution. They were first given six guidelines for a productive group discussion (adapted from a study by Schweiger et al., 1986), as shown in Figure 3A. They then entered an online chatroom in which they could communicate with one another. The chatroom interface was divided into two panels: the Player Decisions Panel and the Communication Panel (Figure 4). The Player Decisions Panel, to the left of the terminal display, presented the *T*_1_ graph choices and explanations of each group or dyad member under a pseudonym (Leda, Triton, Portia, or Sinope) to preserve participant anonymity. In the Communication Panel, to the right of the terminal display, dyad and group members could communicate with one another by typing messages into a text entry field. These messages were posted in the Communication Panel under the group or dyad member's designated pseudonym. A timer in the top right corner of the terminal display showed how much time remained. After 10 minutes had elapsed, one group or dyad member was chosen randomly by the computer to register the group's or dyad's consensus decision.

The procedure at *T*_2_ was different in the individuals condition. Participants in this condition were instructed to reflect on their *T*_1_ decision for 10 minutes, alone. They were instructed to approach this reflection with a skeptical mind, to question their original assumptions, and to consider alternative explanations (Figure 3B). The chatroom interface was once again divided into two panels, this time labeled the Decision Panel and the Reflections Panel. In the Decision Panel, to the left of the terminal display, participants could inspect their *T*_1_ decision and explanation. In the Reflections Panel, to the right of the terminal display, participants were able to record reflections on their *T*_1_ decision. This panel was essentially the same as the Communication Panel for participants in the groups and dyads conditions, except that it was used to record self-reflections, rather than to communicate with group or dyad members. A timer in the top right corner of the terminal display once again indicated how much time remained. After 10 minutes had elapsed, participants were required to indicate once again which graph they deemed to be correct.

After submitting the *T*_2_ decision, all participants completed a post-test questionnaire. Participants in the dyads or groups conditions were asked to choose one of the four CST graphs again, in response to the question; “If the group answer is not what you would have chosen, which answer would you have chosen?”. The post-test questionnaire also contained the climate change knowledge and attitudes questions asked in the individual differences questionnaire at the beginning of the experiment.

The CST took approximately 30 minutes to complete, and the entire experimental session lasted approximately 60 minutes.

## Results

### Time 1

**Figure 5:**
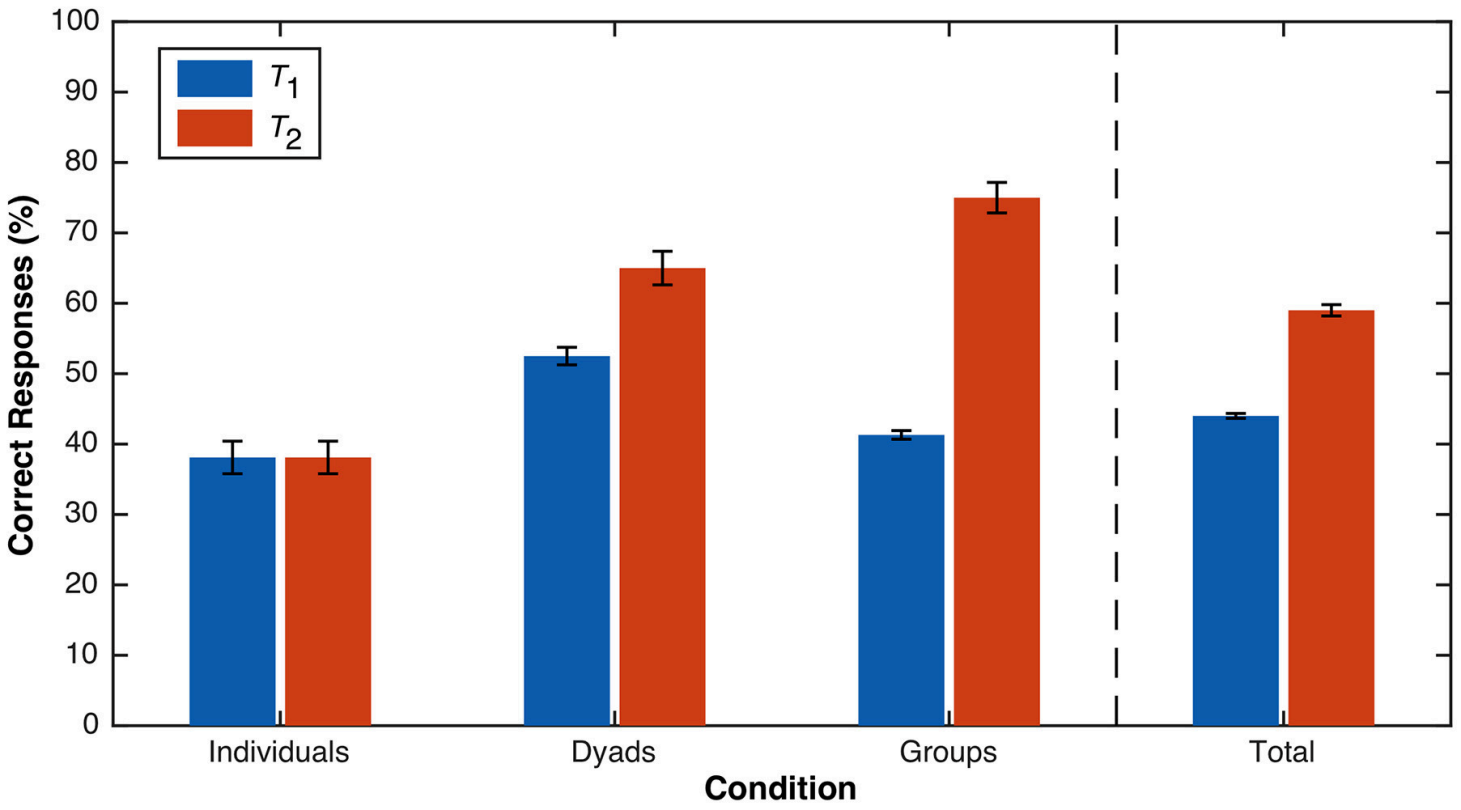
Percentage of correct (Graph D) responses to the CST as a function of time (*T*_1_ vs. *T*_2_) and condition (individuals vs. dyads vs. groups). The bars for *T*_1_ (and *T*_2_ for the individuals condition) represent the percentage of correct individual responses, whereas the bars for the dyads and groups conditions at *T*_2_ represent the proportion of correct dyad and group consensus decisions, respectively. Error bars represent standard errors.

The success rates at *T*_1_ (blue bars; Figure 5) did not differ significantly across the three conditions (χ2_*df = 2*_ = 1.72, *p* = .424, two-sided), and the overall success rate was 44%. This is consistent with the highest previously-reported success rate using the CST (Sterman and Booth-Sweeney, 2002). Table 1 shows the frequency with which participants used various reasoning strategies at *T*_1_ to justify their graph choice. For example, Graph D was frequently accompanied by an explanation correctly describing mass balance principles (88.7% of Graph D responses). Although other strategies were referenced by participants who selected Graph D, every other reasoning strategy was more frequently used to justify an incorrect graph.

**Table 1:** The frequency (%) with which different reasoning strategies were adopted, as a function of Graph A, B, C, and D choices at *T*_*1*_.

**Reasoning strategy and coding criteria**	**Example participant explanation**	**Graph A (*n* = 37)**	**Graph B (*n* = 12)**	**Graph C (*n* = 30)**	**Graph D (*n* = 62)**	**% of total**
**Mass Balance (Correct)** Description indicating awareness ofrelationship between emissions andabsorption flows and the stock ofatmospheric CO_2_; terms such asmass balance, accumulation, rate ofchange.	“It's a mass balance and rates of change situation. For the CO_2_ concentration in the atmosphere tostabilize, you need the rates of emission andabsorption to equal. ”“If emissions are greater than theabsorption, the amount of CO_2_ will increase. If the absorption isgreater than the emissions, the amount of CO_2_ willdecrease.”	0.0	0.0	10.0	88.7	41.1
**Mass Balance (Incorrect)** Description indicating awareness ofrelationship between emissions andabsorption flows and the stock ofatmospheric CO_2_–but misunderstanding the nature of theserelationships.	“In figure D, emissions ended up being the same asabsorption, which causes the concentrationreducing to 0.” “If we keep the same difference between the rate ofemission and absorption, the concentration will bethe same.”	32.4	66.7	56.7	4.8	28.4
**Pattern-Matching** Description mentioning correlations orsimilarity of behavior or patternsamong emissions and atmosphericCO_2_; indication that emissions shouldbe proportional to changes inatmospheric CO_2_.	“As I understand it, there is a direct relationshipbetween CO_2_ emissions and the atmosphericconcentration.” “If atmospheric concentrationincreases, that meansthat CO_2_ emissions will also increase.” “[Graph A] because it rises and then stabilizes.”	59.5	33.3	10.0	1.6	21.3
**Mathematical Reasoning** Using algebraic equations, calculatingratios, or quantifying the absolutevalues of atmospheric concentration,emissions, and/or absorption.	“To achieve the quantity of 420 ppm, should havean increase of 20 ppm. The emission should be only20% higher than the absorption.” “The rate of ppm increase from 1990 to 2025 = 120ppm/125 years = 0.96 ppm/ear. The ratio of GtC toppm is around 4GtC = 1ppm.”	21.6	50.0	16.7	8.1	17.0
**Reasonableness of Trajectories** Indicates belief that the correcttrajectory should reflectbusiness-as-usual or personalpredictions about futureemissions/absorption rates.	“…it would be too idealistic to imply that the changewould be immediate and the decline would be asdrastic as depicted in options B, C, and D” “Withcurrent pressures on countries by the UNFCCC forsetting emission reduction targets, countries willtake drastic measures to reduce their carbon emissions.”	21.6	33.3	20.0	6.5	15.6

*Reasoning strategy was inferred from participants' T_1_ post-decision explanations. The first column lists the possible reasoning strategies, and the coding criteria for those strategies, whilst the second column gives example participant explanations that conform to each strategy. The third through sixth columns show the percentage of participants who chose Graph A, B, C, or D, respectively, and subsequently referenced each reasoning strategy. As any one explanation could refer to more than one reasoning strategy, the values in columns three through six may sum to >100%. The last column shows the percentage of total responses that referenced each reasoning strategy. Again, this sums to >100% because multiple strategies were possible. For inter-rater reliability information, see Supplementary Materials, Section Inter-Rater Reliability for Coding of Reasoning Strategies at T_1_*.

The full coding scheme consisted of five strategies from Sterman and Booth-Sweeney (2007) and their associated coding criteria, plus four additional categories created *post-hoc* to capture reasoning strategies that did not conform to any previously-defined category. The strategies taken from Sterman and Booth-Sweeney (2007) were: pattern-matching, mass balance, technology, sink saturation, and CO_2_ fertilization (for details see Table 7 in Sterman and Booth-Sweeney, 2007). Two additional categories defined in their coding scheme (energy balance and inertia/delays) were not used by any of our participants, and therefore were not included here. Two of the new categories were simply the reverse of the categories identified by Sterman and Booth-Sweeney (2007): mass balance—incorrect (incorrect understandings of mass balance) and technology—reverse (technology will increase emissions, rather than enable emissions reduction). The final two categories, mathematical reasoning, and reasonableness of trajectories, were created by the authors on the basis of an analysis of participants' responses. In this paper, we only report on the five most popular strategies (technology, sink saturation, CO_2_ fertilization, and technology—reverse were used by < 3% of total participants and were therefore excluded from the current analysis).

The literature tends to attribute incorrect answers on the CST to the “pattern-matching” heuristic (e.g., Sterman and Booth-Sweeney, 2007; Sterman, 2008; Cronin et al., 2009). Pattern-matching was indeed the most popular reasoning strategy for participants who selected the typical pattern-matching graph of Graph A (Figure 2A). However, pattern-matching was not the most popular incorrect reasoning strategy overall. As shown in the last column of Table 1, across all responses, incorrect mass balance principles were applied more frequently than pattern-matching. “Mathematical reasoning” and “reasonableness of trajectories” were also common, especially for participants who chose Graph B. The popularity of these strategies suggests that errors on the CST are not exclusively caused by rash, heuristic decisions (i.e., pattern-matching)—even participants who used deliberate and effortful approaches (e.g., unsuccessfully trying to relate CO_2_ emissions with CO_2_ absorption, or calculating ratios) failed to reach the correct answer.

### Time 2

There was more heterogeneity in success rates across conditions at *T*_2_ (orange bars; Figure 5), and the overall success rate of 59% was marginally higher than at *T*_1_. The success rate for dyads (65.0%) did not differ significantly from that for individuals (38.1%) (χ2 _*df* = 1_ = 2.97, *p* = .121, two-sided) or groups (75.0%) (χ2 _*df* = 1_ = 0.48, *p* = .731, two-sided). However, the success rate for groups was significantly higher than for individuals (χ2_*df* = 1_ = 5.67, *p* = .028, two-sided). A more diagnostic set of comparisons involves contrasting the difference in success rates between *T*_1_ and *T*_2_ for each condition, separately. For individuals, there was no change in success rates over time (*p* = 1.00, McNemar, two-sided). For dyads, the success rate of dyad consensus decisions at *T*_2_ (65.0%) was numerically, but not significantly, higher than that of individual dyad member decisions at *T*_1_ (52.5%) (*p* = .125, McNemar, two-sided). Recall that after dyads submitted their consensus decision at *T*_2_, individual dyad members were prompted for the answer they would have chosen, regardless of what the consensus decision was. Again, there was no difference in success rates between answers given by individual dyad members at *T*_1_ and then at this post-test stage (both 52.5%) (*p* = 1.00, McNemar, two-sided). For groups, the success rate of group consensus decisions at *T*_2_ (75.0%) was significantly higher than that of individual group member decisions at *T*_1_ (41.3%) (*p* < .001, McNemar, two-sided). Furthermore, the success rate of individual group members' post-test answers (61.3%) was significantly higher than the success rate of individual group members at *T*_1_ (41.3%) (*p* = .002, McNemar, two-sided). Thus, the group discussion reliably improved CST success rates, while the individual reflection and dyad discussion did not.

Analysing the content of *T*_2_ reflections and discussions (in the same way as analyzing the content of *T*_1_ explanations) sheds light on how groups derived their decision-making advantage over individuals. Despite explicit instructions to “approach this with a skeptical mind,” “question your own assumptions,” and “consider alternative arguments,” 80% of participants in the individuals condition did not type anything during the 10-minute reflection period. Of 21 individuals, only one typed an alternative argument, and only three changed their answers at *T*_2_. Individuals failed to self-reflect, thus preventing them from recognizing their answer was incorrect, or considering why a different answer may be correct. This is consistent with previous research characterizing individuals as “cognitively lazy” decision-makers who rarely challenge an answer that “feels” right (Trouche et al., 2015; Larrick, 2016).

By contrast, all groups entertained at least two reasoning strategies in their group discussions (except one group in which all group members selected Graph D at *T*_1_). Group discussions contained a mean of 2.30 different reasoning strategies, compared to 1.15 for dyad discussions, and 0.24 for individual reflections (χ2_*df* = 2_ = 33.48, *p* < .001, two-sided). Furthermore, we have tentative evidence that group members helped correct other members' faulty reasoning strategies. For example, in one group, one participant's misunderstanding of mass balance principles (“If emission rate gets close to absorption, concentration will decrease below 400[ppm]”) was corrected by two other participants who explained the principle of accumulation (“…but when emissions is greater than absorption, then concentration will increase”). Groups were more likely than individuals (χ2_*df* = 1_ = 23.89, *p* < .001, two-sided) and dyads (χ2_*df* = 1_ = 8.29, *p* = .010, two-sided) to refer to the correct reasoning strategy of mass balance (even if no group member had referenced mass balance in their *T*_1_ explanation). This supports previous findings showing that exposure to diverse perspectives motivates group members to critically evaluate all arguments (Trouche et al., 2015), thus increasing the likelihood that the correct decision will be discussed and judged to be correct (Schulz-Hardt et al., 2006).

The data seem to speak against—but do not rule out—the alternative explanation that groups only benefited from the increased probability of having one member who knew the correct answer. Eight individuals, 15 dyads, and 15 groups contained at least one member who chose Graph D at *T*_1_. If the effect was due merely to the presence of a correct member, we would expect equal performance between dyads and groups, and also for dyads to outperform individuals at *T*_2_ (which they did not, χ2_*df* = 1_ = 2.97, *p* = .121, two-sided). Furthermore, two groups gave the correct consensus decision at *T*_2_, despite having no members who gave the correct decision at *T*_1_ (an example of “process gain,” in which interpersonal interaction between multiple individuals yields an outcome better than that of any single individual, or even the sum of all individuals; Hackman and Morris, 1975).

We were also able to rule out effects of individual differences. Binary logistic regression analyses were conducted to determine whether the various individual difference variables measured at pre-test subsequently predicted performance on the CST at *T*_1_. To satisfy the assumption of a dichotomous dependent variable, CST performance was coded as either incorrect (selecting Graphs A, B, or C) or correct (selecting Graph D). Age, actively open-minded thinking, and supporting the policy of “government regulation of CO_2_ as a pollutant” were the only significant independent predictors (*p* < .05). These variables were subsequently combined into a set of predictors and subjected to a further binary logistic regression analysis. The full model was statistically significant compared to the constant-only model, χ^2^_*df* = 3_ = 21.47, *p* < .001, Nagelkerke's *R*^2^ = 0.19. Prediction accuracy was 61.9% (50.8% for correct responses, 70.5% for incorrect responses). Thus, the full model with these three predictors was barely above chance at predicting correct answers. This poor predictive performance is noteworthy in revealing that performance on the CST is largely immune to the influence of demographic, attitudinal, personality, and cognitive style variables.

Recall that the individual difference questions about climate change knowledge and attitudes were presented again at the post-test phase. CST performance was not significantly predictive of answers to any of these items. However, in light of the third aim of our study, it is worth discussing the answers to the policy preference question, “Which of these comes closest to your view on how we should address climate change?”. At pre-test, 69.1% of participants answered “act-now,” 30.9% answered “go-slow,” and no participant selected the “wait-and-see” option. Excluding the wait-and-see option, answering the CST correctly did not significantly predict post-test responses (χ^2^_*df* = 3_ = 7.19, *p* = .066, two-sided). However, there was an increase in the percentage of individuals who selected “act-now” from pre-test to post-test across all conditions (overall, 80.9% “act-now” at post-test).

## Discussion

Repeated studies employing the CST reveal that individuals systematically underestimate the emissions reduction required to stabilize atmospheric CO_2_ levels. So far, interventions to increase success rates on the CST have been individual-focused and largely ineffective. We sought to examine whether group reasoning could increase success rates on this task. It was predicted that success rates from *T*_1_ to *T*_2_ would (1) not increase for individuals, (2) increase for dyads, and (3) increase for groups to a greater extent than for dyads. The first and third predictions were statistically supported, but there was only qualitative support for the second prediction. The 10-minute group discussions among four participants significantly improved success rates, whereas 10 minutes of individual reflection or dyad discussion did not. By analyzing individual justifications at *T*_1_, and reflections and discussions at *T*_2_, we found that groups benefited from exposure to multiple perspectives and the opportunity to communicate, which facilitated the falsification of incorrect reasoning strategies. We also found that incorrect reasoning strategies were numerous, and not limited to the oft-reported pattern-matching strategy. Lastly, we rejected two alternative explanations—groups did not improve merely due to a size advantage in the number of members who knew the correct response, nor were individual differences in demographics, climate change knowledge, personality, or cognitive style responsible for any given individual's CST success.

There are some potential limitations of the current study that merit consideration. Firstly, with a minimum of 20 cases in each condition as units for the statistical analysis, our experiment may have had insufficient statistical power to detect a significant improvement in success rates for dyads from *T*_1_ to *T*_2_. Using a larger sample size may reveal that the numerical, yet non-significant, increase in success rates observed with our dyad members reflects some real benefit of the dyad discussion. Secondly, the group advantage observed in the current study was obtained using a multiple-choice response format, which is different to the conventional CST procedure in which participants must sketch trajectories of emissions and absorption. It therefore remains open whether the results reported here would generalize to an experimental scenario employing this more complicated response format. It is possible, for example, that the uncharacteristically low rates of susceptibility to the pattern matching heuristic observed in the current study are an artifact of our unorthodox response format. Thus, we may expect higher initial rates of pattern-matching when returning to the original CST procedure, but it remains to be seen whether this would eliminate or attenuate the group advantage witnessed here. Thirdly, our intervention at *T*_2_ in the dyad and group conditions afforded more than merely the opportunity for individuals to communicate with one another—participants were also afforded the chance to read the *T*_1_ explanations of their dyad partner or group members. Although it is our conviction that the opportunity to engage in communication was instrumental to the group decision making advantage, we cannot preclude the possibility that mere exposure to the alternative perspectives of others may confer an advantage in itself, compared to individual reasoning alone. A group condition in which participants are exposed to their group members' *T*_1_ decision explanations—without engaging in any subsequent discussion—would reveal whether mere exposure to multiple perspectives can produce a group benefit. Finally, we could not provide strong evidence for Sterman's (2008) argument that policy-makers' and citizens' deficient mental models of the climate system are responsible for complacent attitudes toward emissions reduction. Answers to the CST did not predict subsequent policy preferences. However, all participants in our sample believed that we must take action against climate change (“act-now” or “go-slow”), even before completing the CST. We were therefore unable to test the hypothesis that participants who answer the CST incorrectly also deny the need for emissions reduction (“wait-and-see”). However, the overall increase in “act-now” responses, relative to “go-slow” responses, from pre-test to post-test implies some diffuse benefit of merely completing the CST. Future studies employing a sample with more heterogeneous pre-existing policy beliefs will provide a stronger test for the hypothesized link between accurate mental models of the CO_2_ system and support for urgent emissions reduction.

Given the abovementioned concerns about the generality of our results, one direction for future work is to determine whether and how our findings generalize to other stock-flow tasks, especially the original CST procedure, in which participants sketch trajectories of emissions and absorption by themselves. Furthermore, although we have shown a benefit on CST performance of reasoning in groups of four members, an additional avenue for future work will be to examine whether this advantage extends to larger groups. On the one hand, we might expect that increasing the number of group members will improve CST performance, because of an increase in information-processing capacity and diversity of perspectives (Cohen and Thompson, 2011; Charness and Sutter, 2012). On the other hand, we might expect that as the group size reaches some critical point, CST performance will begin to decline as the aforementioned benefits of group decision making will be outweighed by the costs of coordinating opinions and resolving disputes within the group (Orlitzky and Hirokawa, 2001; Lejarraga et al., 2014). Identifying the optimal group size for solving the CST will permit more robust recommendations about how group-based practices should be incorporated into the decision-making process.

In closing, we note that the final success rate of group consensus decisions at *T*_2_ (75%) is considerably higher than the success rates previously reported with the CST. The success of our group-based intervention suggests that group-based decision-making may help facilitate the two-step implementation of effective emissions policy. First, crafting appropriate mitigation policy requires comprehensive and accurate decision-making, and our results suggest small groups are best-suited for this task. Second, rallying political and electoral support for such policy requires a well-informed population that comprehends the scale of the emissions problem. The general public presently endorses high levels of belief in anthropogenic climate change, but low levels of concern and urgency about climate change mitigation (Akter and Bennett, 2011; Reser et al., 2012). In order to bridge this gap between what the public believes about the climate change problem (that it is real and caused by human activities), and the solutions they are willing to support (immediate and significant emissions reduction), their mental models must be changed. Group-based programs, whether informal conversations about climate change or formal public education initiatives, could establish the correct mental model and help mobilize support for effective mitigation policy.

## Ethics statement

This study was carried out in accordance with the recommendations of the National Statement on Ethical Conduct in Human Research, Human Ethics Office at the University of Western Australia. The protocol was approved by the Human Ethics Office at the University of Western Australia. All subjects gave written informed consent in accordance with the Declaration of Helsinki.

## Author contributions

All authors contributed to the conception and design of the study. MH created the software for running the experiment and produced the figures for the paper. BX conducted the experimental sessions, performed the data analysis, and wrote the paper. MH and IW edited the paper and reviewed the results.

### Conflict of interest statement

The authors declare that the research was conducted in the absence of any commercial or financial relationships that could be construed as a potential conflict of interest.
