# Supplementary material for: Correct Me if I'm Wrong: Groups Outperform Individuals in the Climate Stabilization Task
Source: Front Psychol. 2018 Nov 30;9:2274. doi: 10.3389/fpsyg.2018.02274 (PMC6284004; doi:10.3389/fpsyg.2018.02274)
Supplement: Supplementary file 1 [file Data_Sheet_1.pdf]

Supplementary material for: "Correct me if I'm wrong: Groups outperform individuals in the Climate Stabilization Task"

Belinda Xie[1,2*], **Mark J. Hurlstone**[1], **and Iain Walker**[3]

[1]School of Psychological Science, University of Western Australia, Crawley, Western Australia 6009, Australia
[2]School of Psychology, University of New South Wales, Sydney, New South Wales 2052, Australia
[3]School of Psychology, University of Canberra, Bruce, Australian Capital Territory 2617, Australia
[*]belinda.xie@unsw.edu.au

Xie et al.[1] examined the performance of groups, dyads, and individuals on the Climate Stabilization Task (hereafter, 'CST').[2] This supplementary document reports additional details about the experiment including the experimental instructions given to participants; the individual differences questionnaire that preceded the CST; and additional details regarding the coding of data and analysis of results. Note that this document is not meant to be self-explanatory; please consult Xie et al.[1] for further information.

1 Individual Differences Questionnaire

A battery of ten scales assessed individual differences in demographics, climate change knowledge and attitudes, personality, and cognitive style. These measures were chosen because previous research indicates that they may influence performance on the CST (e.g., because they influence performance on other stock-flow reasoning tasks). All scales have good psychometric properties, as shown in previous research. The scales were administered in the following fixed but random order.

1.1 The Actively Open-Minded Thinking Scale[6]

Active open-mindedness is an individual's tendency to consider new evidence in relation to a favored belief, and to integrate others' opinions into one's own opinion.[6] This scale was included as open-mindedness is positively related to individual and group performance on various different reasoning tasks[7,8] and the ability to avoid bias by prior beliefs.[9] We tentatively predicted that higher active open-mindedness scores would be associated with better performance on the CST.

Instructions and items: Please indicate how much you agree with each statement, from 1 (*completely disagree*) to 7 (*completely agree*).

1. Intuition is the best guide in making decisions. (R)
2. Changing your mind is a sign of weakness. (R)
3. People should revise their beliefs in response to new information or evidence.
4. One should disregard evidence that conflicts with one's established beliefs. (R)
5. It is important to persevere in your beliefs, even when evidence is brought to bear against them. (R)
6. People should take into consideration evidence that goes against their beliefs.
7. Allowing oneself to be convinced by an opposing argument is a sign of good character.

Scoring: The Actively Open-Minded Thinking Scale yields a composite score which is created by summing the responses to its seven items. Note that items marked (R) are reverse coded prior to scoring.

1.2 Cognitive Reflection Test[10]

This scale assesses an individual's ability to resist reporting the first response that comes to mind.[10] It was included because solving the CST is thought to require the suppression of the intuitive 'pattern-matching' heuristic. Consistent with this notion, it has been shown that Cognitive Reflection Test scores are positively correlated with performance on a kindred stock-flow reasoning task.[11]

Accordingly, it was predicted that higher scores on the Cognitive Reflection Test would be associated with better performance on the CST.

Instructions and items: Please answer the following items:

1. A bat and a ball cost $1.10 in total. The bat costs $1.00 more than the ball. How much does the ball cost (in cents)?

2. In a lake, there is a patch of lily pads. Every day, the patch doubles in size. If it takes 48 days for the patch to cover the entire lake, how long would it take for the patch to cover half of the lake? (in days)

3. If it takes 5 machines 5 minutes to make 5 widgets, how long would it take 100 machines to make 100 widgets? (in minutes)

Scoring: The Cognitive Reflections Test yields a composite score which is the sum of the correct answers to its three items (1. = 5 cents; 2. = 47 days; 3 = 5 minutes).

1.3 Climate Change Knowledge: Perceived[12]

Both perceived and objective climate change knowledge were included because task-relevant knowledge is associated with better performance on some stock-flow tasks,[13] but appears to be unrelated to performance on the CST.[24] We therefore made no specific prediction about the relationship between climate change knowledge and CST performance.

Instructions and items: How much do you feel you know about climate change?

☐ Nothing at all
☐ A little
☐ A moderate amount
☐ A lot

1.4 Climate Change Knowledge: Objective[14]

Instructions and items: Which of these activities cause increases in atmospheric greenhouse gases? (*cause*, *not a cause*, or *don't know*)

1. People heating and cooling homes

2. Use of aerosol spray cans

3. Nuclear power generation

4. People driving their cars

5. Pollution/Emissions from business and industry

6. Use of chemicals to destroy insect pests

7. Destruction of forests

8. Use of coal and oil by utilities and electric companies

9. Depletion of ozone in the upper atmosphere

Scoring: A composite score is created by summing all correct responses. Items 1, 4, 7, and 8 are scored correct if participants respond with "cause", the remaining items are scored correct if participants respond with "not a cause".

1.5 Environmental Worldview[15]

Environmental worldview refers to one's beliefs about humanity's relationships with nature.[15] Those who view the environment as ductile are more likely to support solutions involving government intervention than those who view the environment as elastic,[15] which may affect answers on the CST. We included this measure to rule out the possibility that participants' answers to the CST were based on their political ideology, rather than their stock-flow reasoning.

Instructions and items: Please indicate how much you agree with each statement, from 1 (*strongly disagree*) to 5 (*strongly agree*).

1. The natural environment will become unstable if humans exceed the limits identified by experts.

2. The natural environment is capable of recovering from any damage humans may cause.

3. The natural environment can be managed if there are clear rules about what is allowed.

4. Ultimately, there's nothing individuals can do to manage or change the natural environment.

5. If the balance of the natural environment is upset the whole system will collapse.

6. Conservation and protection is the most rational strategy for managing the natural environment.

7. When pushed beyond the limits identified by experts the natural environment will not recover.

8. We all have a moral obligation to protect the environment and consume fewer resources.

9. There's no point wasting time, energy and resources on trying to manage the natural environment.

10. Individuals should have freedom of choice regardless of the environmental impacts.

11. Human industry and technology has not caused significant damage to the natural environment.

12. Humans can't control what happens in the natural environment.

Scoring: Scores from items 1, 3, 4, 5, 6, and 7 sum to produce the "environment as ductile" scale, while scores from the remaining items sum to produce the "environment as elastic" scale. Both scale scores were used in the individual differences analysis (see section 3.1).

1.6 Need for Cognition[16]

Need for cognition refers to an individual's need to structure situations in meaningful and integrated ways.[16] Individuals high in need for cognition prefer complex over simple tasks[17] and are more likely to discriminate between strong and weak arguments.[18] We predicted that higher scores on the need for cognition scale would be associated with better performance on the CST.

Instructions and items: Please indicate how much you agree with each statement, from 1 (*very strong disagreement*) to 9 (*very strong agreement*). There are no right or wrong answers.

1. I usually end up deliberating about issues even when they do not affect me personally.

2. I try to anticipate and avoid situations where there is likely chance I will have to think in depth about something. (R)

3. I feel relief rather than satisfaction after completing a task that required a lot of mental effort. (R)

4. I prefer my life to be filled with puzzles that must be solved.

5. I really enjoy a task that involves coming up with new solutions to problems.

6. I prefer to think about small, daily projects to long-term ones. (R)

7. Thinking is not my idea of fun. (R)

8. The idea of relying on thought to make my way to the top appeals to me.

9. I would rather do something that requires little thought than something that is sure to challenge my thinking abilities. (R)

10. I find satisfaction in deliberating hard and for long hours.

11. I would prefer complex to simple problems.

12. I only think as hard as I have to. (R)

13. It's enough for me that something gets the job done; I don't care how or why it works. (R)

14. I would prefer a task that is intellectual, difficult, and important to one that is somewhat important but does not require much thought.

15. Learning new ways to think doesn't excite me very much. (R)

16. I like tasks that require little thought once I've learned them. (R)

17. The notion of thinking abstractly is appealing to me.

18. I like to have the responsibility of handling a situation that requires a lot of thinking.

Scoring: The Need for Cognition Scale produces a composite score which is the sum of the responses to its eighteen items. Note that items marked (R) are reverse coded prior to scoring.

1.7 Climate Change Attitudes[19]

We wanted to ensure the CST was a decision-making task and not simply an indirect measure of climate change attitudes. We predicted the absence of a relationship between climate change attitudes and success rates on the CST.

Instructions and items:

1. Do you believe that the earth is getting warmer mostly because of human activity such as burning fossil fuels, or mostly because of natural patterns in the earth's environment?

☐ Entirely caused by natural processes
☐ Mainly caused by natural processes
☐ Partly natural process, partly human activity
☐ Mainly caused by human activity
☐ Entirely caused by human activity
☐ Climate change is not occurring
☐ Don't know
☐ No opinion

2. Which comes closest to your view on how we should address climate change?

☐ Climate change is a serious and pressing problem. We should begin taking steps now even if this involves significant costs.
☐ Climate change should be addressed, but its effects will be gradual, so we can deal with the problem gradually by taking steps that are low in cost.
☐ Until we are sure that climate change is really a problem, we should not take any steps that would have significant economic costs.

3. Would you support the following policy proposals to mitigate global warming? (*yes*, *no*, or *don't know* what this policy means)

1. Shifting subsidies from fossil fuels to renewable energy

2. Tax based on a vehicle's fuel economy

3. A tax on energy used by businesses

4. Mandatory reductions in Australia's greenhouse gas emissions

5. Government regulation of carbon dioxide as a pollutant

6. Increase in vehicle fuel economy standards

Scoring: Responses to items 1 and 2, and items 3.1 to 3.6, were included as separate categorical predictors in the individual differences analysis (see section 3.1).

1.8 Systems Thinking Scale[20]

Systems thinking is a worldview that recognizes cause-effect relationships emerge from complex, dynamic, and nested systems.[20,21] Systems thinking is related to climate change risk perception, support for policies to regulate emissions, and the tendency to consider causal complexity.[21] We predicted that higher scores on the Systems Thinking Scale would be associated with better performance on the CST.

Instructions and items: Please indicate how much you agree with each statement, from 1 (*strongly disagree*) to 7 (*strongly agree*).

1. When I have to make a decision in my life I tend to see all kinds of possible consequences to each choice.

2. Social problems, environmental problems, and economic problems are all separate issues. (R)

3. I like to know how events or information fit into the big picture.

4. Only very large events can significantly change big systems like economies or ecosystems. (R)

5. All the Earth's systems, from the climate to the economy, are interconnected.

6. Everything is constantly changing.

7. Adding just one more, small farm upstream from a lake can permanently alter that lake.

8. When a boom or a crash happens in part of the world's economy, it is because someone intentionally planned or designed for it to run that way. (R)

9. Ultimately, we can break all problems down to what is simply right and wrong. (R)

10. The Earth, including all its inhabitants, is a living system.

11. Rules and laws should not change a lot over time. (R)

12. If I make plans and control my behavior I can accurately predict how my life will unfold. (R)

13. Seemingly small choices we make today can ultimately have major consequences.

14. My health has nothing to do with what is happening in the world. (R)

15. It is possible for a community to organize into a new form that was not planned or designed by an authority or government.

Scoring: The Systems Thinking Scale produces a composite score which is the sum of the responses to its fifteen items. Note that items marked (R) are reverse coded prior to scoring.

1.9 Kimchi-Palmer Figures Task[22,23]

This task assesses an individual's processing style. Processing style is a content-free way of perceiving the world perceptually and conceptually, in which global processes attend to the overall picture ('gestalt'), whereas local processes attend to component parts.[11] One study found that accuracy in one stock-flow task was greater for global processors compared to local processors,[23] but another study found Kimchi-Palmer-Figures Task score to be unrelated to stock-flow performance.[11] We therefore made no specific prediction about the relationship between global/local processing style scores and performance on the CST.
Instructions and items: You will now be shown a series of three figures (see Figure 1 for the complete set of figures); one figure at the top and two below it. Please click the figure at the bottom that you think most resembles the figure at the top. Answer quickly with your immediate, first choice. There are no right answers.

Scoring: Each item contains a global processing item and a local processing item. The amount of items for which participants choose the global processing item is summed to produce the "global processing index", while the amount of items for which participants choose the local processing item is summed to produce the "local processing index". Both indices were incorporated as predictors in the individual differences analysis (see section 3.1).

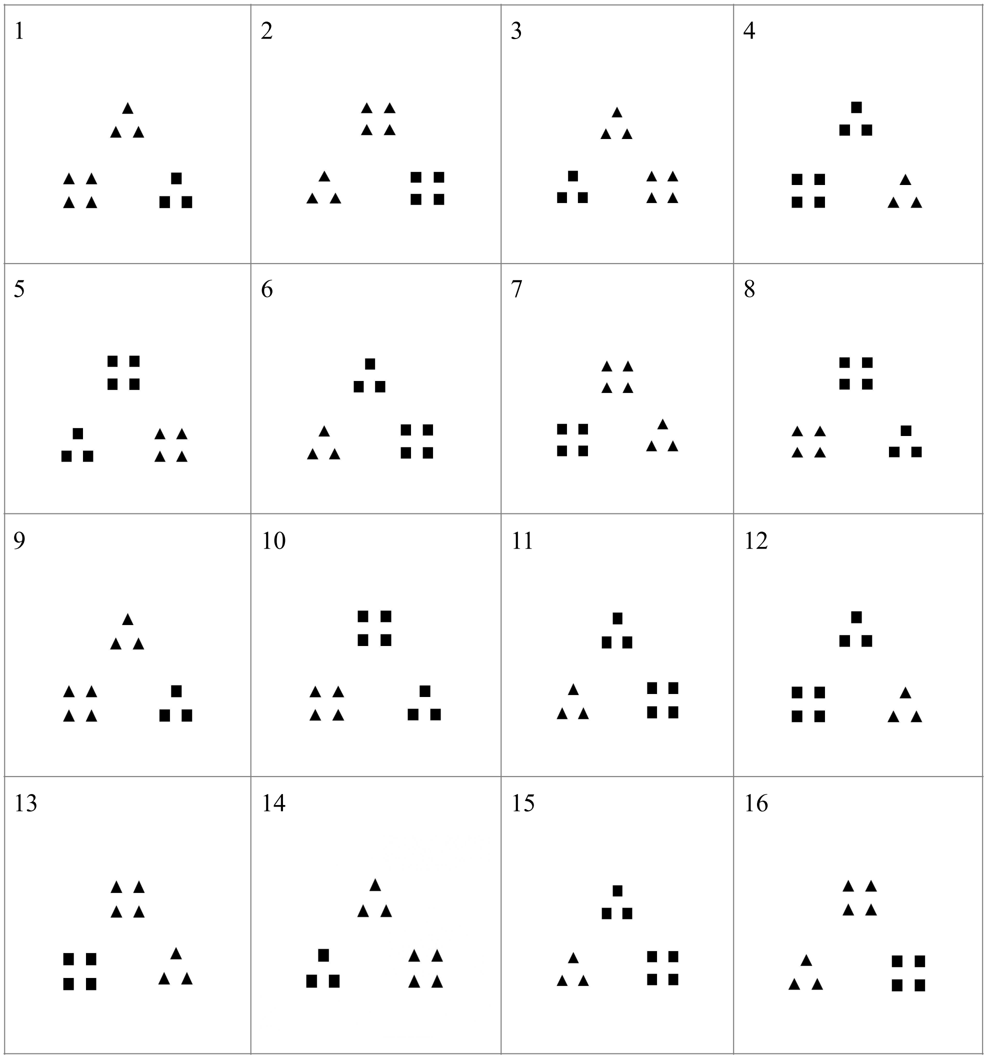

Figure 1. Stimuli used in the Kimchi-Palmer-Figures Task.[22] Participants are presented with one reference figure that is either a triangle or square, made up of three or four smaller triangles or squares. Participants select one of two response figures below the reference figure. For example, in item 1, the bottom left figure represents the local processing item, as the individual shapes are the same as the individual shapes in the reference figure (triangles), while the bottom right figure represents the global processing item, as the overall shape is the same as the overall shape of the reference figure (triangle).

2 Instructions for CST

Welcome to Today's Study!

In this study, you will learn about the two factors that influence the concentration of carbon dioxide (hereafter, 'CO_2') in the atmosphere. This refers to the amount of CO_2 gas in the air surrounding the earth. We will then ask you to imagine a scenario in which atmospheric CO_2 concentration reaches a certain level in the future. Your task will be to determine the level of CO_2 emissions, in order for this scenario to be realized.

What is CO_2?

CO_2 is a clear gas that occurs naturally in the Earth's atmosphere. CO_2 can be thought of in terms of its atmospheric concentration, and the rates of emissions and absorption. These three measures are defined below in Figure 1 (Figure 2):

What Affects Atmospheric CO_2 Concentration?

Although human and non-human factors can each increase and decrease atmospheric CO_2 concentration, human activity is the dominant factor that increases CO_2, and natural processes are the dominant factors that decrease CO_2.

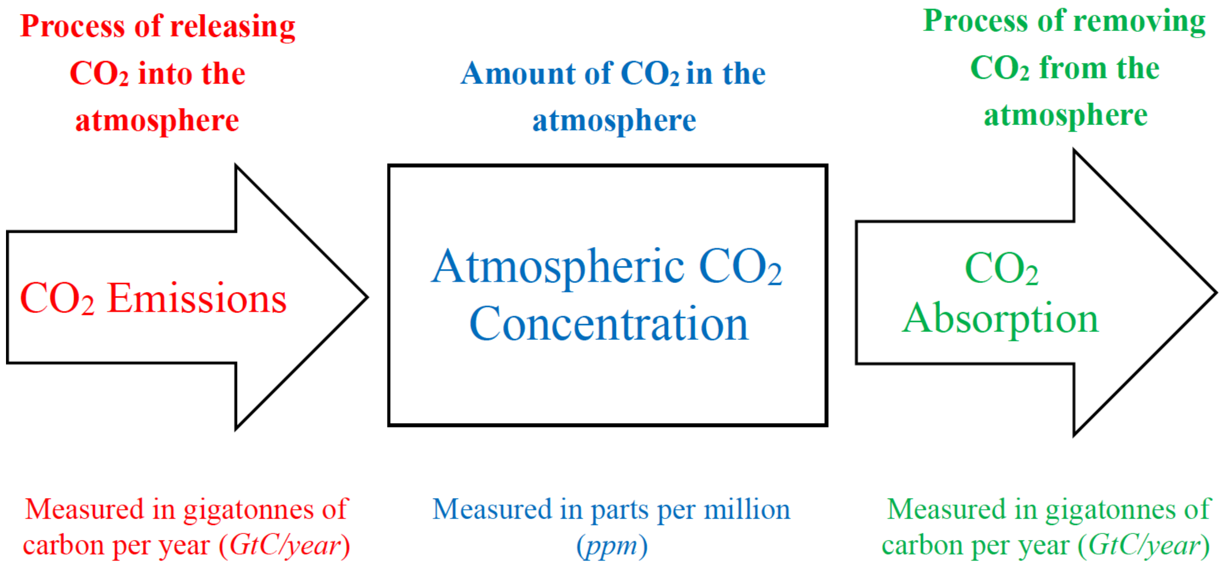

Figure 2. Summary of emissions, concentration, and absorption of CO_2.

- CO_2 emissions resulting from human activity increase atmospheric CO_2 concentration. Emissions have been growing since the Industrial Revolution. Fossil fuel burning, certain industrial processes, and land-use changes currently input around 8GtC/year.
- Natural absorption processes decrease atmospheric CO_2 concentration, by removing CO_2 from the atmosphere. Oceans, plants, and other factors currently remove about 4GtC/year.
- The current rate of emissions is currently twice the current rate of absorption—this means we are generating more CO_2 than the Earth can remove. As a result, atmospheric CO_2 concentration has increased, from pre-Industrial Revolution levels of 280ppm, to 400ppm today.

The current values of emissions, absorption, and atmospheric CO_2 concentration are displayed below in Figure 2 (Figure 3):

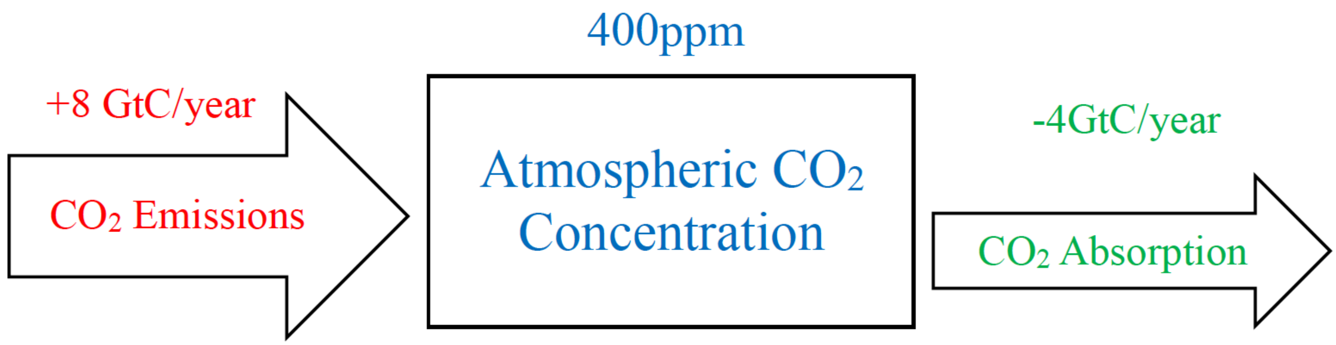

Figure 3. Atmospheric CO_2 concentration is increased by CO_2 emissions and decreased by CO_2 absorption. The rate of CO_2 emissions is currently twice as large as the rate of CO_2 absorption.

Decision Making Task

Now consider a scenario in which atmospheric CO_2 concentration gradually rises from its current level of 400ppm to stabilize at 420ppm by the year 2100, as shown in Figure 3 (Figure 4).

The four graphs in Figure 4 (Figure 5) show absorption and emissions projected from 1900 to 2100. Absorption is the same, but emissions from today until 2100 differ in each graph. **On the computer to your right, please indicate which of these graphs would lead to the scenario depicted in Figure 3 (Figure 5) where atmospheric CO_2 stabilizes at 420ppm by 2100.**

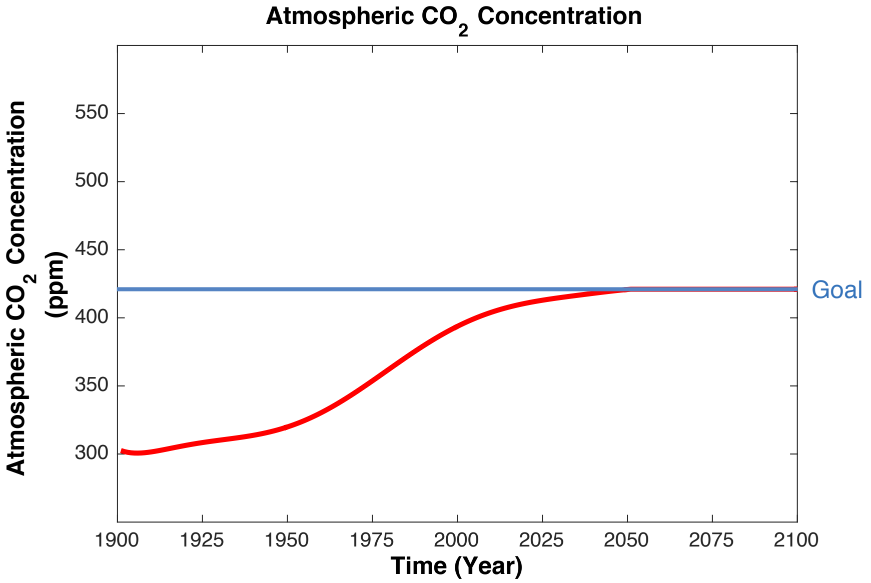

Figure 4. Atmospheric CO_2 concentration stabilizes at 420ppm by 2100. The *y*-axis shows atmospheric CO_2 concentration in ppm and the *x*-axis shows time in years.

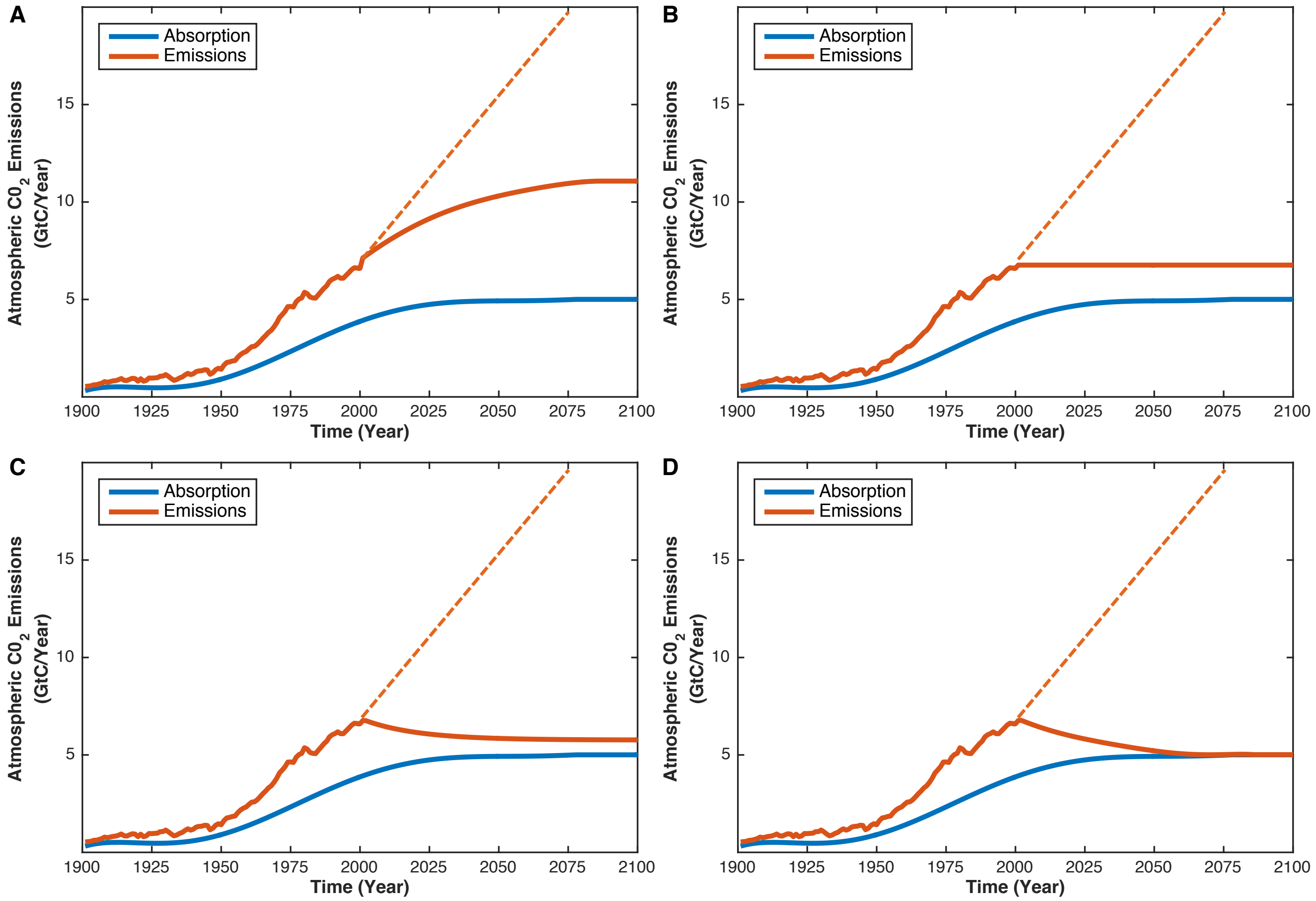

Figure 5. Four possible emissions pathways. The *y*-axis shows absorption and emissions in GtC/year and the *x*-axis shows time in years.

3 Inter-Rater Reliability for Coding of Reasoning Strategies at T_1

The explanations provided by all participants at T_1 were coded to identify the reasoning strategies used to justify each graph choice. Any single explanation could include more than one reasoning strategy. The explanations were coded independently by the first author, and again by a second rater who was not involved in data collection and was naïve to the study aims and hypotheses. All discrepancies between the first and second raters were resolved by discussion, and the final coding results were mutually agreed upon by both raters. Table 1 shows, for each reasoning strategy, the reliability between: the final consensus coding and the first rater's original coding (column 1); the final consensus coding and the second rater's original coding (column 2); and the first rater's and second rater's original codings (column 3). Note that Table 1 includes four reasoning strategies, namely 'technology' (the belief that technology will reduce emissions or enhance absorption), 'technology (reverse)' (the belief that technology will increase emissions), 'sink saturation' (the belief that absorption may fall due to sink saturation), and 'CO_2 fertilization' (the belief that absorption may rise due to enhanced plant growth or some other variable) that were not referenced in Xie et al.[1] because because these strategies were very infrequently observed.

Strategy	Consensus × Rater 1	Consensus × Rater 2	Rater 1 × Rater 2
Pattern-Matching	.820	.404	.293
Mass Balance (Correct)	.911	.956	.867
Mass Balance (Incorrect)	.688	.879	.557
Mathematical Reasoning	.923	.542	.428
Reasonableness of Trajectories	.808	.536	.366
Technology	1.00	.657	.657
Technology (Reverse)	1.00	.563	.563
Sink Saturation	.797	.561	.320
CO_2 Fertilization	.563	.743	.797

Table 1. The Cohen's κ coefficients between: the final consensus codings and the first rater's original codings; the final consensus codings and the second rater's original codings; the first rater's original codings and the second rater's original codings (all $p < .001$).

References

1. Xie, B, Hurlstone, M. J., & Walker, I. Correct me if I'm wrong: A group decision-making intervention improves reasoning in the climate stabilization task *Manuscript submitted for publication* (2018).
2. Sterman, J. D. & Booth-Sweeney, L. Understanding public complacency about climate change: Adults' mental models of climate change violate conservation of matter. *Climatic Change* **80**, 213–238 (2007).
3. Greiner, B. Subject pool recruitment procedures: Organizing experiments with ORSEE. *Journal of the Economic Science Association* **1**(1), 114–125 (2015).
4. Fischbacher, U. z-Tree: Zurich toolbox for ready-made economic experiments. *Experimental Economics* **10**, 171–178 (2007).
5. Schweiger, D. M., Sandberg, W. R. & Ragan, J. W. Group approaches for improving strategic decision making: A comparative analysis of dialectical inquiry, devil's advocacy, and consensus. *Acad. Manage. J.* **29**, 51–71 (1986).
6. Haran, U., Ritov, I. & Mellers, B. A. The role of actively open-minded thinking in information acquisition, accuracy, and calibration. *Judgment and Decision Making* **8**, 188–201 (2013).
7. Boschetti, F., Richert, C., Walker, I., Price, J. & Dutra, L. Assessing attitudes and cognitive styles of stakeholders in environmental projects involving computer modelling. *Ecol. Model.* **247**, 98–111 (2012).
8. Homan, A. C. et al. Facing differences with an open mind: Openness to experience, salience of intragroup differences, and performance of diverse work groups. *Acad. Manage. J.* **51**, 1204–1222 (2008).
9. West, R. F., Toplak, M. E. & Stanovich, K. E. Heuristics and biases as measures of critical thinking: Associations with cognitive ability and thinking dispositions. *J. Educ. Psychol.* **100**, 930 (2008).
10. Frederick, S. Cognitive reflection and decision making. *J. Econ. Perspect* **19**, 25–42 (2005).
11. Weinhardt, J. M., Hendijani, R., Harman, J. L., Steel, P. & Gonzalez, C. How analytic reasoning style and global thinking relate to understanding stocks and flows. *J. Oper. Manage.* **39–40**, 23–30 (2015).
12. Malka, A., Krosnick, J. A. & Langer, G. The association of knowledge with concern about global warming: Trusted information sources shape public thinking. *Risk. Anal.* **29**, 633–647, (2009).

13. Rounds, J., & Armstrong, P. I. Men and things, women and people: A meta-analysis of sex differences in interests. *Psychol. Bull.* **135**, 859–884 (2009).

14. O'Connor, R. E., Bord, R. J. & Fisher, A. Risk perceptions, general environmental beliefs, and willingness to address climate change. *Risk. Anal.* **19**, 461–471 (1999).

15. Price, J. C., Walker, I. A. & Boschetti, F. Measuring cultural values and beliefs about environment to identify their role in climate change responses. *J. Environ. Psychol.* **37**, 8–20 (2014).

16. Petty, R. E., Cacioppo, J. T. Kao, C. F. The efficient assessment of need for cognition. *J. Pers. Assess.* **48**, 306–307 (1984).

17. Cacioppo, J. T., & Petty, R. E. The need for cognition. *J. Pers. Soc. Psychol.* **42**, 116–131 (1982).

18. Petty, R. E., Cacioppo, J. Morris, K. Effects of need for cognition on message evaluation, recall, and persuasion. *J. Pers. Soc. Psychol.* **45**, 805–818 (1983).

19. Martin, J. F. Improving understanding of climate change dynamics using interactive simulations Unpublished Masters Thesis, Massachusetts Institute of Technology (2008).

20. Davis, A. C. & Stroink, M. L. The relationship between systems thinking and the new ecological paradigm. Syst. Res. Behav. Sci. **33**(4), 575–586 (2016).

21. Lezak, S. B., & Thibodeau, P. H. Systems thinking and environmental concern. *J. Environ. Psychol.* **46**, 143–153 (2016).

22. Kimchi, R., & Palmer, S. E. Form and texture in hierarchically constructed patterns. *J. Exp. Psychol. Human.* **8**, 521–535 (1982).

23. Fischer, H., & Gonzalez, C. Making sense of dynamic systems: How our understanding of stocks and flows depends on a global perspective. *Cognitive. Sci.* **40**, 1–17 (2015).

24. Moxnes, E., & Saysel, A. K. Misperceptions of global climate change: Information policies. *Working Papers in System Dynamics* **1**, 1–51 (2004).

25. Sterman, J. D. Does formal system dynamics training improve people's understanding of accumulation? *Syst. Dynam. Rev.* **26**, 316–334 (2010).

26. Browne, C. Compston, P. In Reinventing Life on a Shrinking Earth: Proceedings of the 33rd International Conference of the System Dynamics Society (Cambridge, USA, 2015).
